# Effect of perioperative esketamine use on emergency delirium in children undergoing tonsillectomy and adenoidectomy: a systematic review and meta-analysis of randomized controlled trials

**DOI:** 10.3389/fmed.2025.1505408

**Published:** 2025-01-29

**Authors:** Junfeng Liu, Jinming Liu, Hong Sun, Xue Cheng, Chunhui Wang, Daoyun Lei, Chao Han

**Affiliations:** ^1^Department of Anesthesiology, The Affiliated Yixing Hospital of Jiangsu University, Yixing, Jiangsu, China; ^2^Department of Anesthesiology, Zhongda Hospital Southeast University, Nanjing, Jiangsu, China

**Keywords:** emergence delirium, esketamine, meta-analysis, perioperative medicine, systematic review

## Abstract

**Background:**

Emergence delirium(ED) is a common postoperative complication in children undergoing tonsillectomy and adenoidectomy under general anesthesia. There is no high-quality evidence on the relationship between esketamine and ED. The systematic review and meta-analysis was performed to investigate the effect of perioperative esketamine use on ED in children undergoing tonsillectomy and adenoidectomy.

**Method:**

We searched Embase, The Cochrane Library, PubMed, MEDLINE, Web of Science, China National Knowledge Infrastructure (CNKI), WanFang, VIP, and SinoMed from inception to 1 September, 2024. Two evaluators identified randomized controlled trials comparing perioperative use of esketamine with placebo or other drugs in children undergoing tonsillectomy and adenoidectomy. Incidence of ED was the primary outcome of the study. Data synthesis was performed by using Review Manager 5.4 software.

**Results:**

Twenty-three relevant studies involving a total of 1,996 children were identified. Perioperative use of esketamine reduced the incidence of ED in children undergoing tonsillectomy and adenoidectomy (RR = 0.33, 95% CI: [0.25, 0.44], *p* < 0.00001, *I*^2^ = 0%). Scores of ED were lower in the esketamine group than in the control group (SMD = -1.20, 95% CI: [−1.56,-0.84], *p* < 0.00001, *I*^2^ = 88%). Children in the esketamine group have lower postoperative pain scores (SMD = -0.51, 95% CI: [−0.80,-0.39], *p* < 0.00001, *I*^2^ = 74%). Esketamine was also associated with a lower incidence of adverse events (RR = 0.75, 95% CI: [0.57, 0.99], *p* = 0.04, *I*^2^ = 62%). We also found that the use of esketamine reduced the length of stay in the post-anesthetic care unit (PACU) but had no effect on the time to extubation.

**Conclusion:**

Perioperative use of esketamine could significantly reduce the incidence of ED in children undergoing tonsillectomy and adenoidectomy. However, the optimal dose and timing of esketamine administration for preventing ED remains to be explored.

**Systematic review registration:**

https://www.crd.york.ac.uk/PROSPERO/display_record.php?RecordID=558560, PROSPERO: CRD42024558560.

## 1 Introduction

Emergence delirium(ED) is a clinically recognized condition that often occurs during the recovery phase of anesthesia and is characterized by agitation, confusion, and restlessness (1). ED is a common perioperative complication in children with a prevalence of approximately 10–80% (2). ED is self-limiting and lasts in typically 15–30 min, but the long-term postoperative cognitive changes of ED in children are unknown (3). Hazards of emergence delirium include wound dehiscence, accidental removal of intravenous infusion tubes or drains, and falling out of bed (4). ED may also cause harm to healthcare workers and family members caring for the child, increase the difficulty of postoperative care, increase the incidence of postoperative complications, and be detrimental to the child’s postoperative recovery (1, 5).

Tonsillectomy and adenoidectomy are the most common surgeries performed on children, with more than 500,000 annually in the United States (6). Pediatric adenoidectomies and tonsillectomies are characterized by short operating times, severe stress response, and high levels of postoperative pain. Currently, general anesthesia with opioids combined with propofol and sevoflurane is widely used for tonsillectomy and adenoidectomy. However, sevoflurane causes a high incidence of delirium during the awakening period, and opioids have the disadvantage of respiratory and circulatory depression (7, 8). After tonsillectomy and adenoidectomy, children often experience complications such as pain, bleeding, nausea, and vomiting. ED can lead to an increased incidence of these complications (9). Therefore, it is necessary to find an appropriate anesthesia plan to reduce the occurrence of delirium during the awakening period and provide comfortable medical treatment for children.

Esketamine is the dextro isomer of ketamine, and its anesthetic and analgesic effects are mainly achieved by non-competitive antagonism of N-methyl-D-aspartic acid (NMDA) receptors (10). Esketamine is an intravenous anesthetic with analgesic properties that can be safely used for the induction and maintenance of general anesthesia and postoperative analgesia. Esketamine has the advantages of good analgesic effect, slight respiratory depression, and inhibition of inflammatory response (11). Subanesthetic doses of esketamine can exert antidepressant effects and improve postoperative cognitive dysfunction (12, 13). Esketamine can also reduce the use of opioid analgesics and even antagonize opioid-induced respiratory depression (14, 15).

Although a number of clinical trials have been conducted to study the relationship between esketamine and ED in pediatrics under general anesthesia, they are all small-sample studies and still lack high-quality evidence. In this study, we investigated the effect of esketamine on delirium during the awakening period after tonsillectomy and adenoidectomy by meta-analysis method to provide a clinical reference.

## 2 Materials and methods

### 2.1 Overview and registration

This meta-analysis was conducted following the recommendations of the Preferred Reporting Items for Systematic reviews and Meta-Analyses (PRISMA) statement and registered in the International Prospective Register of Systematic Reviews (PROSPERO) database (registration number CRD42024558560).

### 2.2 Search strategy

The literature search was conducted on Embase, The Cochrane Library, PubMed, MEDLINE, Web of Science, CNKI, WanFang, VIP, and SinoMed from inception to 1 September 2024. Four key search terms (‘Emergence Delirium’, ‘Esketamine’, ‘Tonsillectomy or Adenoidectomy’ and ‘Chindren’), with varition, were used and combined using Boolean operators. There were no restrictions on language, gender, sample size, or geographic location during the literature search. Supplementary Table 1 lists the search strategies adapted for each database. Articles that may be eligible by reviewing the reference lists of retrieved studies will be included in this meta-analysis (16, 17).

### 2.3 Inclusion criteria

To assess the eligibility of the acquired studies for the meta-analysis, we adopted the following criteria: (1) Population: children undergoing tonsillectomy and adenoidectomy under general anesthesia, (2) Intervention: perioperative intravenous administration of esketamine, (3) Comparison: placebo or other drugs, (4) Outcomes: development of emergence delirium, (5) Study design: randomized controlled trials, (6) statistical methods used correctly, and (7) complete data.

### 2.4 Exclusion criteria

Exclusion criteria were: (1) duplication of published literature, (2) failure to provide valid data or missing data, (3) non-RCT studies such as reviews and animal experiments, and (4) low quality of literature. Included studies were assessed using the Cochrane risk of bias tool. Studies with high risk of bias for randomization or allocation concealment were judged to be of low quality and excluded (18).

### 2.5 Study selection

Records from searches were imported into an EndNote library (EndNote 20) and duplicate studies were removed. The remaining records were transferred into an Excel spreadsheet (Microsoft). Articles were screened by 2 independent reviewers who evaluated the article title, abstract, and full text. Studies that did not meet the established inclusion criteria were excluded. Disagreements between two reviewers regarding the inclusion of studies were resolved through discussion or consultation with a third reviewer.

### 2.6 Risks of bias assessment

Two reviewers independently assessed the included studies using the Cochrane risk-of-bias tool. The Cochrane tool was used to assess the possibility of different biases across the included randomized controlled trials, including selection bias, implementation bias, measurement bias, follow-up bias, reporting bias, and other biases (18). Each bias of the studies was categorized as low risk, high risk, and unclear risk. Disagreements settled in consultation with a third reviewer.

### 2.7 Data extraction

Two reviewers independently extracted data items from the included studies. Information collected included first author, year of publication, age, gender, sample size, mode of induction of anesthesia, study design, experimental group intervention, control group intervention, incidence of emergence delirium, severity of emergence delirium, level of pain, time to extubation, length of stay in the post-anesthetic care unit (PACU), and incidence of adverse events. Disagreements between the two reviewers regarding the data were resolved by consulting a third reviewer.

### 2.8 Statistical analysis

In this study, data synthesis was performed by using Review Manager 5.4 software (Cochrane Collaboration; Oxford, UK). Meta-analysis of categorical variables was performed by Mantel–Haenszel (M-H) statistics to calculate the risk ratio (RR) and 95% confidence interval (CI). Meta-analysis of continuous variables was performed by Inverse-Variance (I-V) statistics to calculate the mean difference (MD), standard mean difference (SMD), and 95% confidence interval. When the units and scales of the outcome indicators were the same (such as time to extubation and the length of stay in PACU), MD was used to interpret the results; conversely, SMD was used to interpret the results (such as delirium score and pain score). The results of the 3 studies (19–21) were presented as medians with interquartile ranges. We transformed them into means ± standard deviation (SD) by a reported methodology (22, 23). The *I*^2^ statistic was used to assess statistical heterogeneity between pooled data. *I*^2^ < 50% indicated that study heterogeneity was relatively small, in which case a fixed effects model (FEM) was used to synthesize the data. In contrast, *I*^2^ ≥ 50% indicated that study heterogeneity was relatively large, in which case a random effects model (REM) was used to synthesize the data. All tests were two-tailed test and were defined as statistically significant when *p* < 0.05.

We performed a subgroup analysis to assess whether the relationship between esketamine application and ED was modified by clinical characteristics. The subgroup plan included (1) dose of esketamine administration: ≥ 0.5 mg/kg or < 0.5 mg/kg; (2) timing of perioperative esketamine administration: before anesthesia, during anesthesia (at the time of induction, induction combined with intraoperative maintenance) and at the end of surgery; and (3) type of drug in the control group: saline or blank control, opioid anesthetic drug control, and other anesthetic drug control.

In addition, we assessed publication bias by funnel plots when at least 10 studies reported on the primary outcome measure (24). Sensitivity analysis was performed to explore the impact of study quality on the overall results using successive exclusion of included individual studies.

## 3 Results

### 3.1 Search results and study characteristics

**Figure 1:**
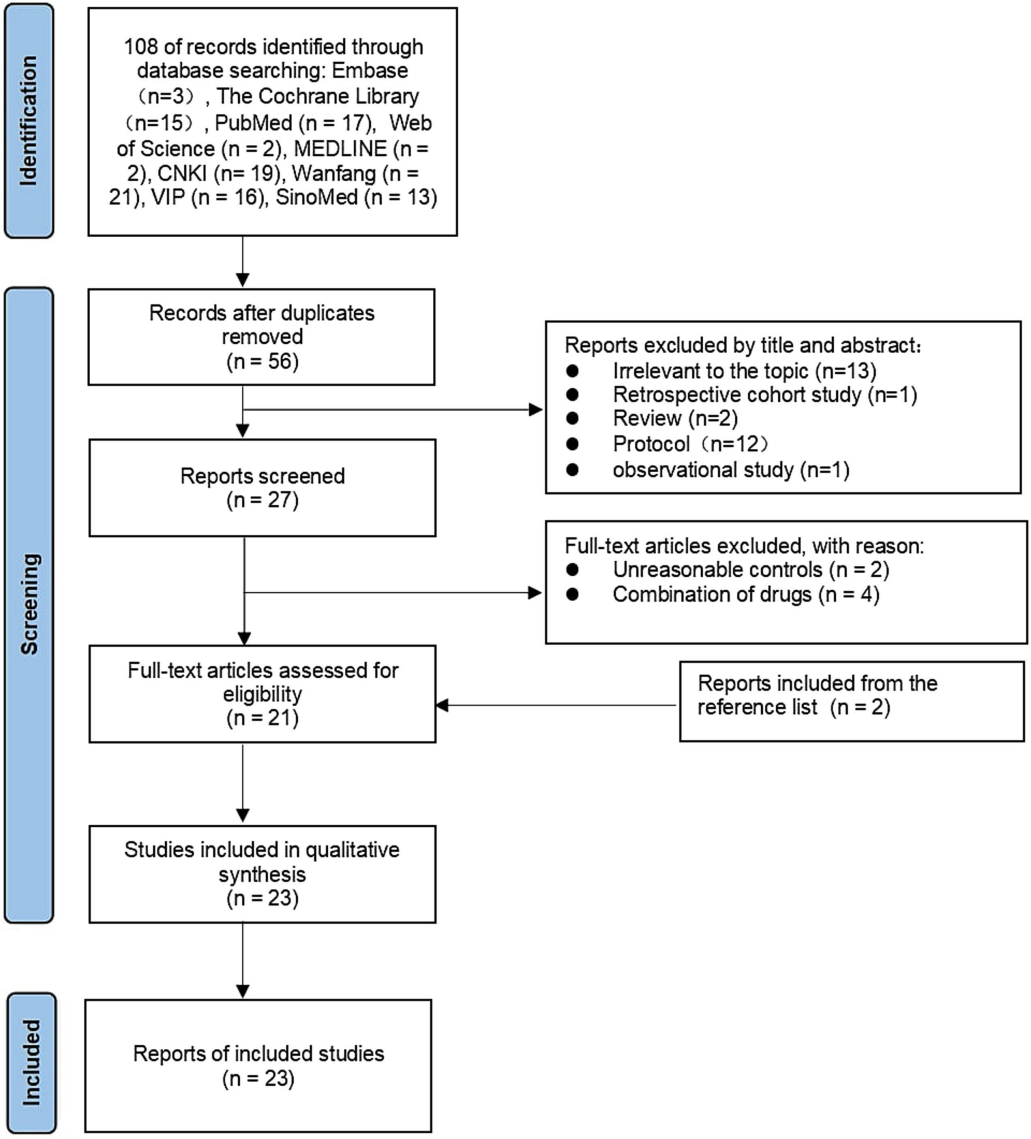
Flow chat.

A total of 108 potentially relevant articles were initially identified from the 9 databases, 52 articles were removed due to duplication, and the remaining 56 studies were screened. We excluded 29 articles due to insufficient relevance based on the title and abstract. The characteristics of the excluded studies are shown in the PRISMA diagram (Figure 1). 27 studies were included in the systematic review, 23 of which were further included in the meta-analysis.

In the 23 studies included, esketamine was administered intravenously. The dose range was 0.15 mg/kg to 1 mg/kg, and the time of administration included before anesthesia, during anesthesia (maintained at induction or in combination with induction), and at the end of surgery (dosage and time of administration in specific studies were recorded in detail in Table 1).

**Table 1:** Characteristics of studies.

Study ID	Age (year)^α^	Gender (male/female)	Procedures	Anesthesia induction	Sample size	Type	Experiment group	Control group	Outcome	Tool
Cao 2022 (25)	3 ~ 10	23/27	Tonsillectomy and adenoidectomy	1-2 mg/kg propofol	E: *n* = 25 C: *n* = 25	RCT	1 mg/kg esketamine (induction of anesthesia) and 0.5–1 mg/kg/h esketamine (maintenance of anesthesia)	2ug/kg fentanyl (induction of anesthesia) and 6-12ug/kg/h remifentanil (maintenance of anesthesia)	ACDEF	PADE, FLACC Watcha
Chen 2022 (29)	E:7.33 ± 0.42; C:7.41 ± 0.32	NA	Tonsillectomy and adenoidectomy	2.5 mg/kg propofol	E: *n* = 30 C: *n* = 30	RCT	0.15 mg/kg esketamine (after induction of anesthesia)	Equivalent volume of saline (after induction of anesthesia)	ACDF	PADE FLACC
Chen 2023(a) (19)	E:5.0 ± 1.4; C:5.4 ± 1.4	E:33/21 C:32/22	Tonsillectomy and/or adenoidectomy	8% sevoflurane	E: *n* = 54 C: *n* = 54	RCT	0.2 mg/kg esketamine (at the end of surgery)	Equivalent volume of saline (at the end of surgery)	ACDEF	PAED CHEOPS
Chen 2023(b) (30)	E:6.53 ± 1.42; C:6.79 ± 1.50	E:58/45 C:52/51	Tonsillectomy or adenoidectomy	8% sevoflurane and 1-2 mg/kg Propofol	E:*n* = 103 C:*n* = 103	RCT	0.5-1 mg/kg esketamine (induction of anesthesia)	2ug/kg remifentanil (induction of anesthesia)	ACDE	PADE FLACC
Chen 2024 (31)	E:5.9 ± 1.0; C:6.1 ± 1.2	E:18/12 C:16/14	Adenoidectomy	Inhaling 8% sevoflurane and giving 1–2 mg/kg propofol after loss of consciousness	E: *n* = 30 C: *n* = 30	RCT	0.5-1 mg/kg esketamine (postoperative analgesia)	0.01 mg/kg hydromorphone (postoperative analgesia)	BDEF	RSS
Cui 2023 (26)	E:7.19 ± 1.20; C:7.25 ± 1.23	E:26/24 C:28/22	Tonsillectomy or adenoidectomy	3 mg/kg propofol	E: n = 50 C: *n* = 50	RCT	0.3 mg/kg esketamine (pre-anesthesia)	Equivalent volume of saline (pre-anesthesia)	ABCDF	PADE FLACC
Jin 2022 (32)	E:5.4 ± 1.6; C:5.1 ± 1.2	E:18/12 C:19/11	Adenoidectomy	1-2 mg/kg propofol	E: *n* = 30 C: *n* = 30	RCT	0.5 mg/kg esketamine (induction of anesthesia)	0.25ug/kg sufentanil (induction of anesthesia)	BCD	RSS VAS
Jin 2024 (33)	E:7.49 ± 0.63; C:7.60 ± 0.66	E:28/12 C:26/14	Tonsillectomy and adenoidectomy	1 mg/kg propofol	E: *n* = 40 C: *n* = 40	RCT	0.25 mg/kg esketamine (induction of anesthesia) and 0.5 mg/kg/h esketamine (maintenance of anesthesia)	2ug/kg fentanyl (induction of anesthesia) and 6ug/kg/h remifentanil (maintenance of anesthesia)	BE	PAED
Li 2022 (17)	E:4.6 ± 1.0; C:4.5 ± 1.3	E:22/18 C:21/19	Tonsillectomy	3 mg/kg propofol	E: *n* = 40 C: *n* = 40	RCT	0.25 mg/kg esketamine (at the end of surgery)	Equivalent volume of saline (at the end of surgery)	BDE	RSS
Li 2024 (34)	E:6.65 ± 1.29; C:6.34 ± 1.53	E:38/31 C:42/27	Tonsillectomy and adenoidectomy	2.5 mg/kg propofol	E: *n* = 69 C: *n* = 69	RCT	0.5 mg/kg esketamine (pre-anesthesia)	0.25 mg/kg dexmedetomidine (pre-anesthesia)	ACDE	PADE FLACC
Liu 2023(a) (16)	E:5.15 ± 2.59; C:5.54 ± 2.60	E:34/29 C:37/24	Adenoidectomy	4 mg/kg propofol	E: *n* = 63 C: *n* = 61	RCT	1 mg/kg esketamine (induction of anesthesia)	2ug/kg fentanil (induction of anesthesia)	CE	FLACC
Liu 2023(b) (35)	E1:7.2 ± 1.9; E2:7.8 ± 2.4; C:6.5 ± 1.8	E1:13/17 E2:14/16 C:14/16	Tonsillectomy and adenoidectomy	2.5 mg/kg propofol	E1:*n* = 30 E2:*n* = 30 C: *n* = 30	RCT	E1:0.3 mg/kg esketamine (induction of anesthesia) E2:0.5 mg/kg esketamine (induction of anesthesia)	Equivalent volume of saline (pre-anesthesia)	BD	RSS
Peng 2022 (36)	E:3.91 ± 1.21; C:4.17 ± 1.31	E:26/19 C:29/16	Tonsillectomy and adenoidectomy	3 mg/kg propofol	E: *n* = 45 C: *n* = 45	RCT	0.5 mg/kg esketamine (pre-anesthesia)	Equivalent volume of saline (pre-anesthesia)	ACDF	PADE RSS FLACC
Shen 2023 (37)	E:9.23 ± 2.04; C:9.12 ± 2.43	E:14/16 C:17/13	Tonsillectomy and adenoidectomy	3 mg/kg propofol	E: *n* = 30 C: *n* = 30	RCT	0.5 mg/kg esketamine (pre-anesthesia)	blank control	ACEF	PAED FLACC
Shi 2024 (27)	E:5.21 ± 0.96; C:5.16 ± 0.92	E:33/27 C:32/28	Adenoidectomy	2.5 mg/kg propofol	E: *n* = 60 C: *n* = 60	RCT	0.5 mg/kg esketamine (induction of anesthesia)	blank control	ACDF	Malviya VAS
Wang 2022 (38)	E:8.1 ± 2.7; C:8.0 ± 2.6	E:23/26 C:25/23	Tonsillectomy and adenoidectomy	3 mg/kg propofol	E: *n* = 49 C: *n* = 48	RCT	0.3 mg/kg esketamine (pre-anesthesia)	Equivalent volume of saline (pre-anesthesia)	ABCDE	Aono FLACC
Wang 2023 (39)	E:5.79 ± 0.82; C:5.83 ± 0.77	E:26/24 C:27/23	Tonsillectomy and adenoidectomy	1-2 mg/kg propofol	E: *n* = 50 C: *n* = 50	RCT	1 mg/kg esketamine (induction of anesthesia)	2ug/kg fentanil (induction of anesthesia)	ACDEF	PAED FLACC
Wu 2023 (42)	1 ~ 4	E:21/10 C:21/9	Tonsillectomy and/or adenoidectomy	1-3 mg/kg propofol	E: *n* = 31 C: *n* = 30	RCT	0.5 mg/kg esketamine (pre-anesthesia)	0.2 mg/kg remimazolam (pre-anesthesia)	BD	PAED RSS
Xiang 2023 (40)	E:6.05 ± 0.72; C:5.93 ± 0.58	E:29/31 C:33/27	Tonsillectomy	2.5 mg/kg propofol	E: *n* = 60 C: *n* = 60	RCT	0.25 mg/kg esketamine (after anesthesia induction)	Equivalent volume of saline (pre-anesthesia)	ACDF	PAED FLACC
Xu 2023 (21)	2 ~ 8	E:28/21 C:32/17	Tonsillectomy	2-3 mg/kg propofol	E: *n* = 49 C: *n* = 49	RCT	0.3 mg/kg esketamine (induction of anesthesia) and 0.3 mg/kg/h esketamine (maintenance of anesthesia)	Equivalent volume of saline (induction and maintenance of anesthesia)	ACDE	PAED FLACC
Yu 2023 (41)	E:4.88 ± 1.08; C:5.22 ± 1.07	E:32/18 C:37/13	Tonsillectomy and/or adenoidectomy	2.5 mg/kg propofol	E: *n* = 50 C: *n* = 50	RCT	0.5 mg/kg esketamine (pre-anesthesia)	0.1 mg/kg midazolam (pre-anesthesia)	ABCDE	PAED FLACC
Zhao 2022 (20)	E:4.9 ± 0.9; C:4.5 ± 1.1	E:22/13 C:18/17	Tonsillectomy and/or adenoidectomy	2-3 mg/kg propofol	E: *n* = 35 C: *n* = 35	RCT	0.25 mg/kg esketamine (at the end of surgery)	0.5ug/kg dexmedetomidine (At the beginning of surgery)	ACDEF	PEAD RSS FPS-R
Zhu 2022 (28)	E1:5.9 ± 2.2; E2:5.8 ± 2.1; C:5.8 ± 2.1	NA	Tonsillectomy and adenoidectomy	3 mg/kg propofol	E1:*n* = 30 E2:*n* = 30 C: *n* = 30	RCT	E1:0.5 mg/kg esketamine (induction of anesthesia) and 0.5 mg/kg/h esketamine (maintenance of anesthesia) E2:0.75 mg/kg esketamine (induction of anesthesia) and 0.75 mg/kg/h esketamine (maintenance of anesthesia)	2ug/kg fentanyl (induction of anesthesia) and 6-12ug/kg/h remifentanil (maintenance of anesthesia)	ACDE	PAED FLACC

α Presented as experiment group vs contrrol group.

A, Severity of emergence delirium; B, Incidence of emergence delirium; C, Pain score; D, Incidence of adverse reactions; E, Time to extubation; F, PACU stay time; PADE, pediatric anesthesia emergence delirium; FLACC, face, legs, activity, cry, consolability, RSS: Ramsay sedation scale; FPS-R, Wong-Baker faces pain scale revision.

### 3.2 Risk of bias assessment

**Figure 2:**
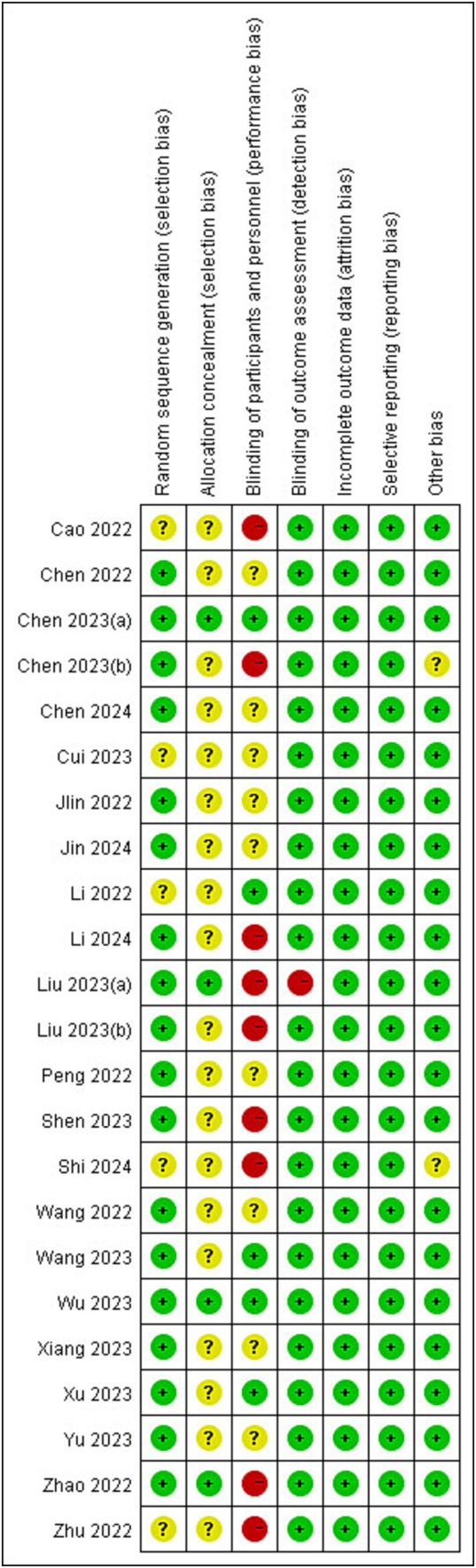
Risks of bias of the included studies. Risk of bias. Green: low risk; yellow: some concern; red: high risk.

**Figure 3:**
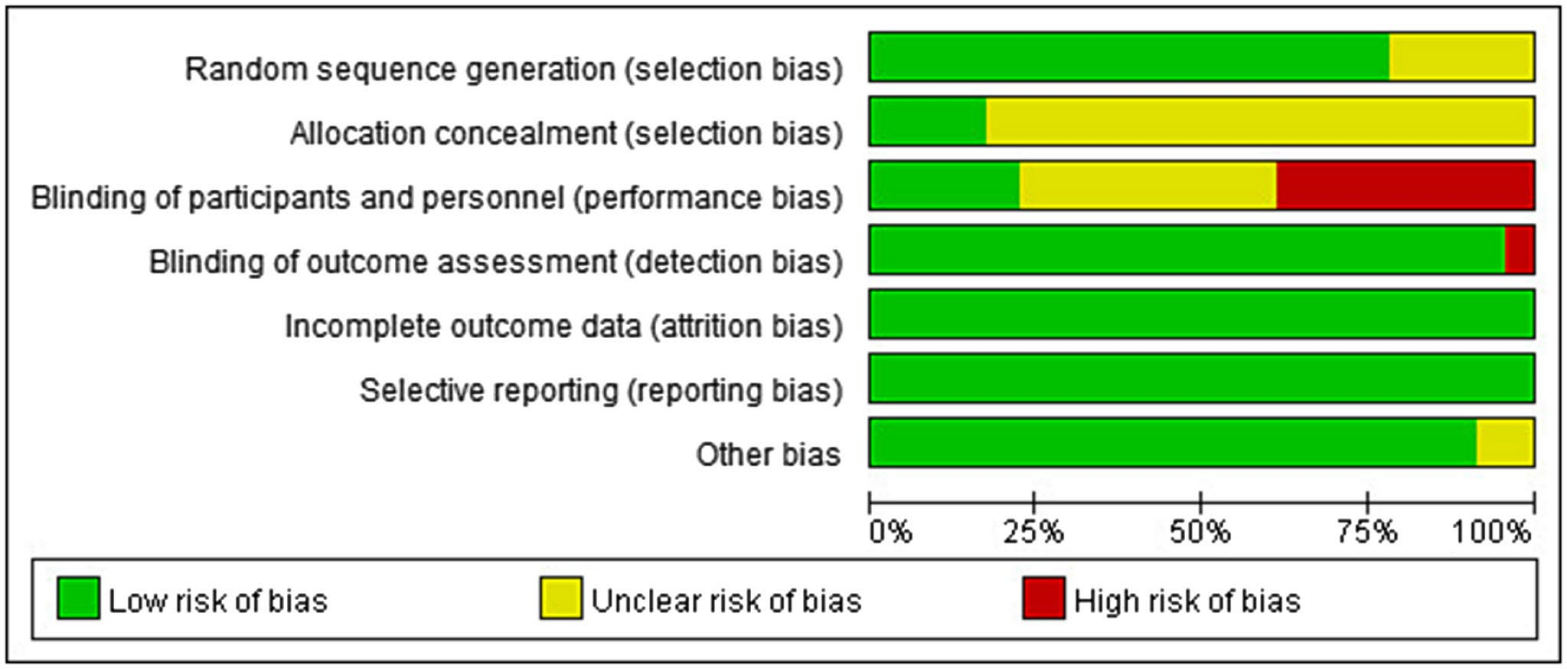
Risk of bias graph.

The risks of bias in individual studies were presented in Figure 2. As for the domain of randomization, 5 RCTs (17, 25–28) were lack of information about randomized methods and 19 RCTs (17, 21, 25–41) were lack of information about allocation concealment. In the domain of intended intervention, 9 RCTs (16, 20, 25, 27, 28, 30, 34, 35, 37) were rated high risk due to specialized intervention methods and 9 RCTs (26, 29, 31–33, 36, 38, 40, 41) were rated unclear risk due to a lack of information on blinding. One RCT (16) was rated high risk in the domain of measurement bias because the researchers know the subgroups. All RCTs were rated low risk in follow-up bias and reporting bias. Regarding other biases, one RCT (30) was rated unclear risk because control group information was not detailed (Figure 3).

### 3.3 Primary outcome

The main indicator of this study is the incidence of emergence delirium. According to different control groups, different doses of esketamine, and different administration times, the corresponding subgroup analysis was carried out. The results of the subgroup analysis were presented in Table 2.

**Table 2:** Subgroup analysis of the incidence of emergence delirium.

Subgroups	Number of studies	Sample size	P for *Q* test	*I*^2^ (%)	Effect mode	RR (95% CI)	P for *Z* test
Overall	13	1,113	0.96	0	FE	0.33 [0.25, 0.44]	<0.00001
Control							
Saline	7	663	0.97	0	FE	0.35 [0.25, 0.49]	<0.00001
Opioids	3	220	0.56	0	FE	0.28 [0.13, 0.58]	0.0006
Other anesthetic drugs	3	230	0.29	18	FE	0.34 [0.20, 0.60]	0.0002
Dose							
Esketamine≥0.5 mg/kg	7	520	0.79	0	FE	0.38 [0.24, 0.60]	<0.0001
Esketamine<0.5 mg/kg	7	593	0.91	0	FE	0.31 [0.22, 0.43]	<0.00001
Time							
Before anesthesia	4	317	0.40	0	FE	0.31 [0.15, 0.63]	0.001
During anesthesia	5	478	0.93	0	FE	0.36 [0.25, 0.52]	<0.00001
At the end of the surgery	4	318	0.81	0	FE	0.31 [0.19, 0.49]	<0.00001

CI, confidence interval; RR, risk ratio; FE, fixed effect.

#### 3.3.1 Overall summary

**Figure 4:**
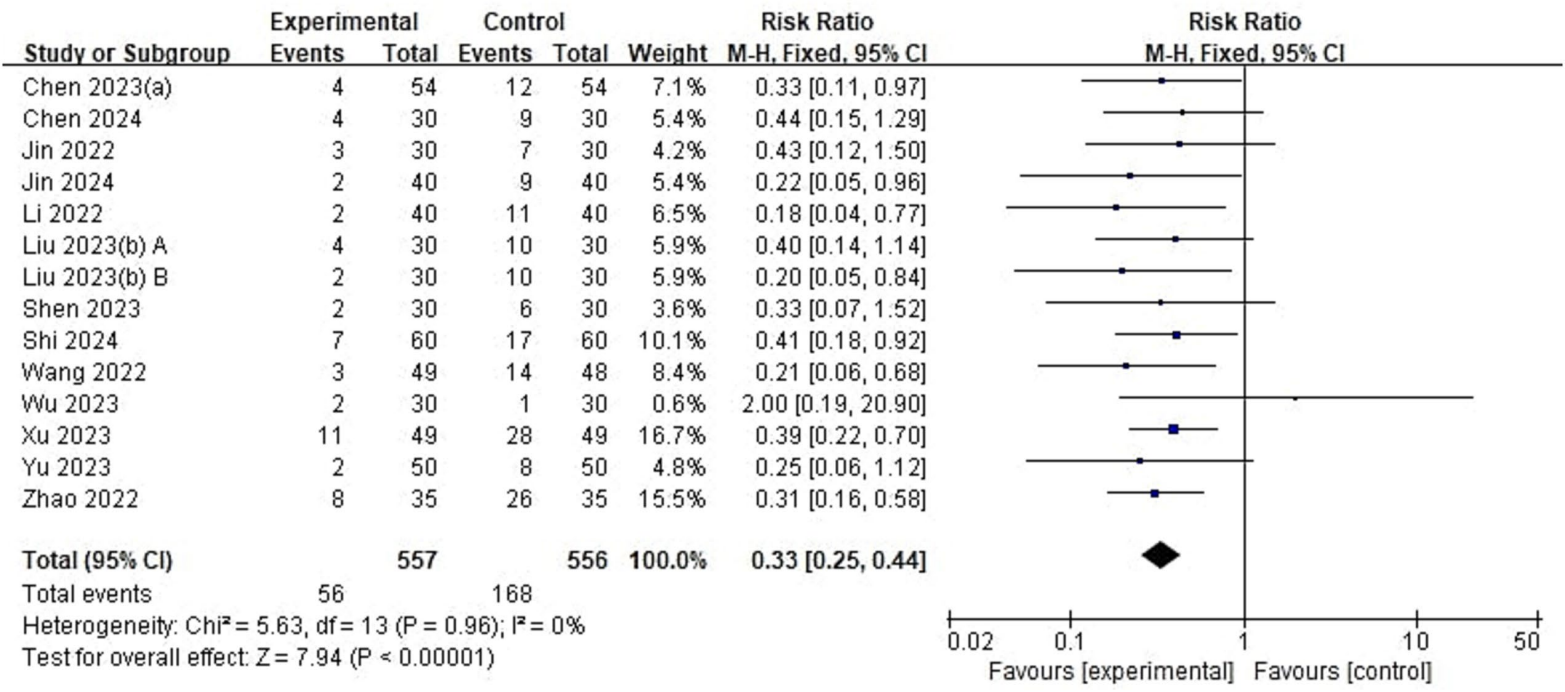
Forest plot comparing the risk of emergence delirium between esketamine and control groups. M-H, Mantel–Haenszel; CI, confidence interval.

Of 23 included studies, 13 reported the incidence of emergence delirium (Figure 4). The incidence of ED in pediatrics treated with esketamine perioperatively was significantly lower than that in the control group (RR = 0.33, 95% CI: [0.25, 0.44], *p* < 0.00001, *I*^2^ = 0%, 13 trials, 1,113 participants).

#### 3.3.2 Subgroup analysis according to the control drug

The control group in 7 studies (17, 19, 21, 27, 35, 37, 38) was saline or blank. These studies showed the incidence of ED in pediatrics treated with esketamine perioperatively was significantly lower than that in pediatrics treated with saline or the blank control (RR = 0.35, 95% CI: [0.25, 0.49], *p* < 0.00001, *I*^2^ = 0%, 7 trials, 663 participants). 3 studies (31–33) indicated pediatrics using esketamine have a lower incidence of emergence delirium compared with opioids (RR = 0.28, 95% CI: [0.13, 0.58], *p* = 0.0006, *I*^2^ = 0%, 3 trials, 220 participants). In the other 3 studies (20, 41, 42), the control groups were other anesthetic drugs including remimazolam, midazolam, and dexmedetomidine. Pediatrics using esketamine also have a lower incidence of emergence delirium compared with other anesthetic drugs (RR = 0.34, 95% CI: [0.20, 0.60], *p* = 0.0002, *I*^2^ = 18%, 3 trials, 230 participants). These results suggest that esketamine could reduce the incidence of ED compared with different control groups, and there were no significant differences between subgroups (*p* = 0.86, Supplementary Figure 1).

#### 3.3.3 Subgroup analysis according to dose of esketamine

The dose of esketamine ≥0.5 mg/kg was the anesthetic dose group and the dose of esketamine <0.5 mg/kg was the subanaesthetic dose group. Anesthetic doses of esketamine were given in 7 studies (27, 31, 32, 35, 37, 41, 42). The incidence of ED was significantly lower in the anesthetic dose group compared with the control group (RR = 0.38, 95% CI: [0.24, 0.60], *p* < 0.0001, *I*^2^ = 0%, 7 trials, 520 participants). Subanaesthetic doses of esketamine were given in 7 studies (17, 19–21, 33, 35, 38). The incidence of emergence delirium was also significantly lower in the low-dose esketamine group compared with the control group (RR = 0.31, 95% CI: [0.22, 0.43], *p* < 0.00001, *I*^2^ = 0%, 7 trials, 593 participants). These results suggest that different doses of esketamine could reduce the incidence of ED, and there were no significant differences between subgroups (*p* = 0.48, Supplementary Figure 2).

#### 3.3.4 Subgroup analysis according to different administration times

Depending on the time of administration of esketamine, these studies were classified as administered before anesthesia, during anesthesia, and at the end of surgery. Esketamine given before anesthesia reduced the incidence of ED compared with the control group (RR = 0.31, 95% CI: [0.15, 0.63], *p* = 0.001, *I*^2^ = 0%, 4 trials, 317 participants). Esketamine administrated during anesthesia reduced the occurrence of emergence delirium compared with the control group (RR = 0.36, 95% CI: [0.25, 0.52], *p* < 0.00001, *I*^2^ = 0%, 5 trials, 478 participants). Pediatrics who were given esketamine at the end of surgery had a lower incidence of emergence delirium compared with the control group (RR = 0.31, 95% CI: [0.19, 0.49], *p* < 0.00001, *I*^2^ = 0%, 4 trials, 318 participants). These results suggest that esketamine administration at different times could reduce the incidence of ED, and there were no significant differences between subgroups (*p* = 0.87, Supplementary Figure 3).

### 3.4 Secondary outcome

Secondary outcomes of this study included delirium scores, pain scores, time to extubation, length of stay in the PACU, and incidence of adverse events (Table 3).

**Table 3:** Analysis of secondary outcome.

Secondary outcome	Number of studies	Sample size	P for *Q* test	*I*^2^ (%)	Effect mode	RR/SMD/MD (95% CI)	P for *Z* test
Delirium scores	14	1,319	<0.00001	88	RE	−1.20 [−1.56, −0.84]	<0.00001
Pain scores	16	1,563	<0.00001	74	RE	−0.51[−0.80, −0.39]	<0.00001
Time to extubation	11	1,072	<0.00001	95	RE	0.01 [−1.44, 1.46]	0.99
Length of stay in the PACU	12	1,025	<0.00001	95	RE	0.28 [0.13, 0.58]	0.07
Adverse events	22	2,142	0.0001	62	RE	0.75 [0.57, 0.99]	0.04

CI, confidence interval; RR, risk ratio; SMD, standard mean difference; MD, mean difference; RE, random effects.

#### 3.4.1 Delirium scores

**Figure 5:**
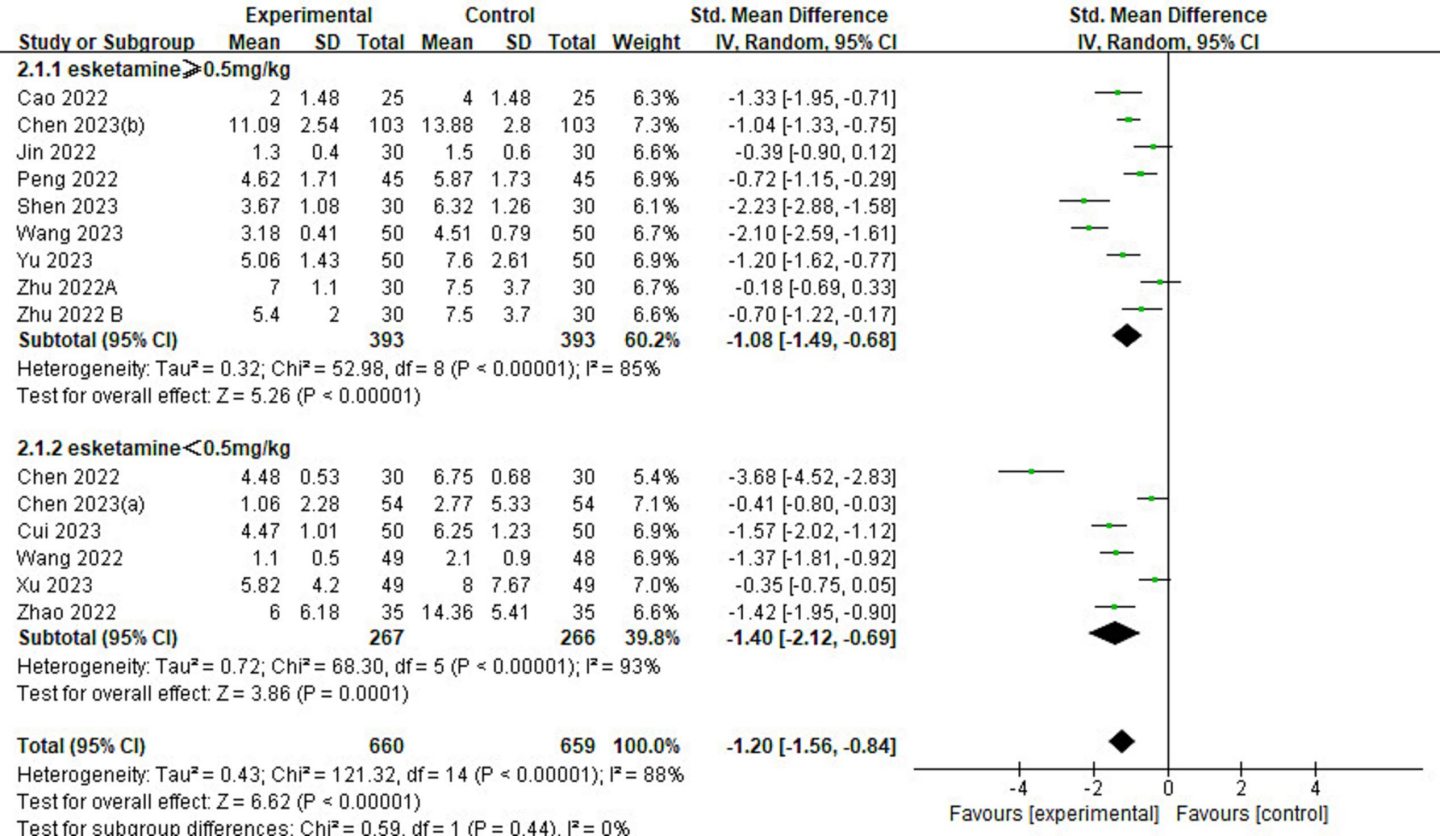
Forest plot comparing the delirium scores between esketamine and control groups. IV, Inverse Variance; CI, confidence interval.

Of the 23 included studies, 14 provided details regarding delirium scores (Figure 5). Analysis results showed that the delirium score in the esketamine group was lower than that in the control group (SMD = −1.20, 95% CI: [−1.56,-0.84], *p* < 0.00001, *I*^2^ = 88%, 14 trials, 1,319 participants), indicating that esketamine was beneficial in reducing the severity of postoperative delirium in children.

#### 3.4.2 Pain scores

**Figure 6:**
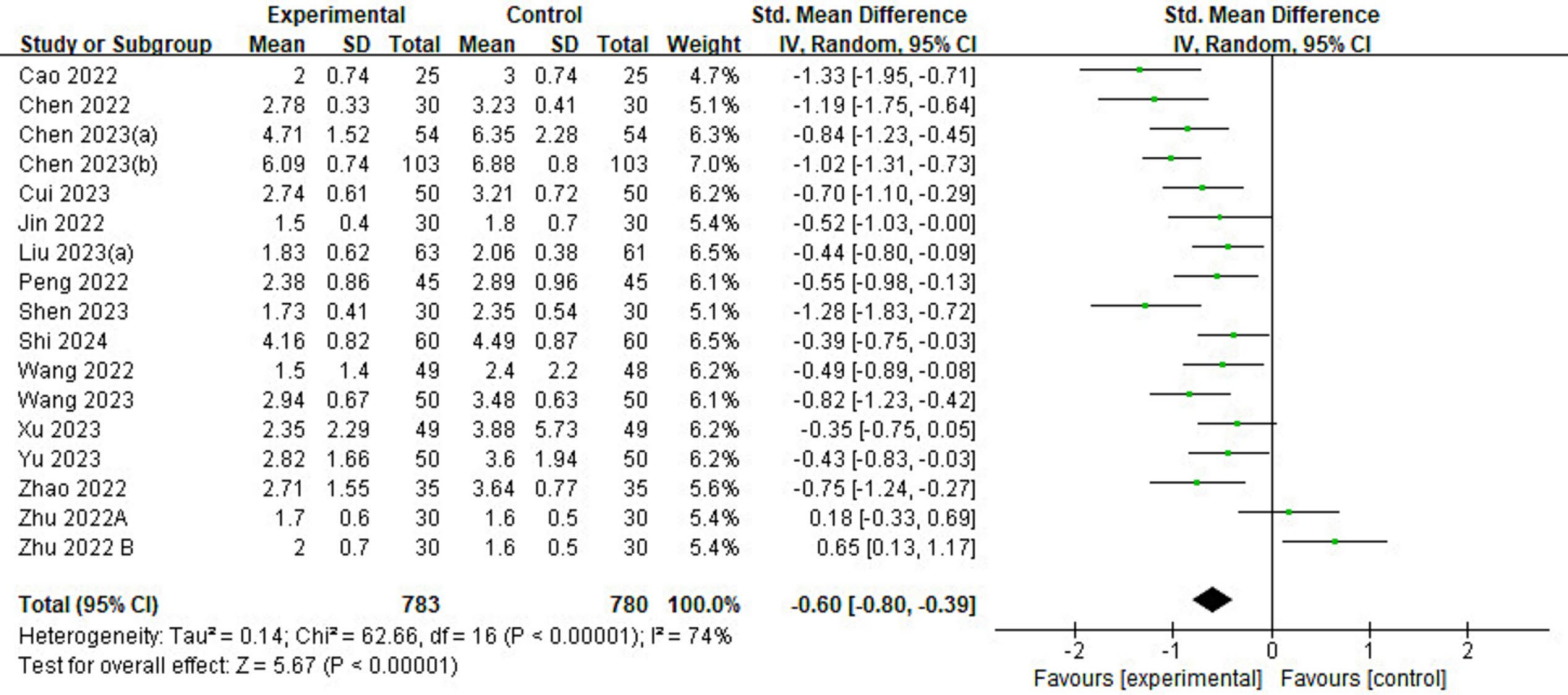
Forest plot comparing the pain scores between esketamine and control groups. IV, inverse variance; CI, confidence interval.

Of the 23 included studies, 16 reported information on pain scores (Figure 6). Pain scores in the esketamine group were lower than those in the control group (SMD = −0.51, 95% CI: [−0.80,-0.39], *p* < 0.00001, *I*^2^ = 74%, 16 trials, 1,563 participants), indicating that esketamine had a positive effect on postoperative pain reduction relief in children.

#### 3.4.3 Time to extubation

**Figure 7:**
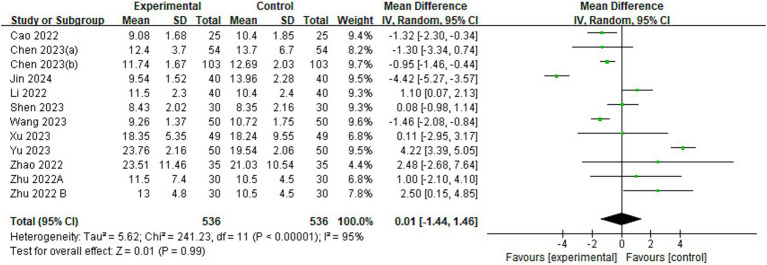
Forest plot comparing the time to extubation between esketamine and control groups. IV, Inverse Variance; CI, confidence interval.

Of the 23 included studies, 11 provided data on time to extubation (Figure 7). The results showed no significant difference in extubation time between esketamine and control groups (MD = 0.01, 95% CI: [−1.44, 1.46], *p* = 0.99, *I*^2^ = 95%, 11 trials, 1,072 participants), implying that esketamine had little effect on postoperative extubation time in children.

#### 3.4.4 Length of stay in the PACU

**Figure 8:**
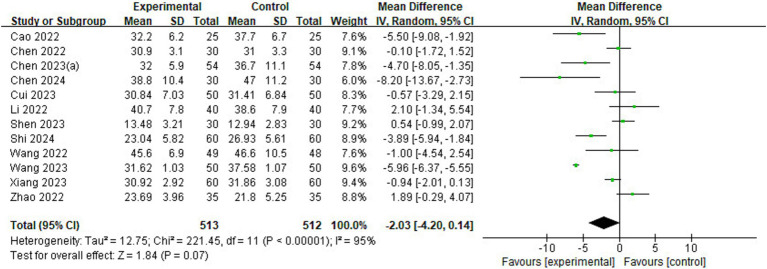
Forest plot comparing the length of stay in the PACU between esketamine and control groups. IV, Inverse Variance; CI, confidence interval; PACU, post-anesthetic care unit.

Of the 23 included studies, 12 reported information on length of stay in the PACU (Figure 8). The analysis found that children in the esketamine group had a shorter stay in the PACU than the control group (MD = −2.03, 95% CI: [−4.20, 0.14], *p* = 0.07, *I*^2^ = 95%, 12 trials, 1,025 participants), and although the *p* value was close to the significance level, it still showed a trend that esketamine may shorten the length of stay in the PACU.

#### 3.4.5 Adverse events

**Figure 9:**
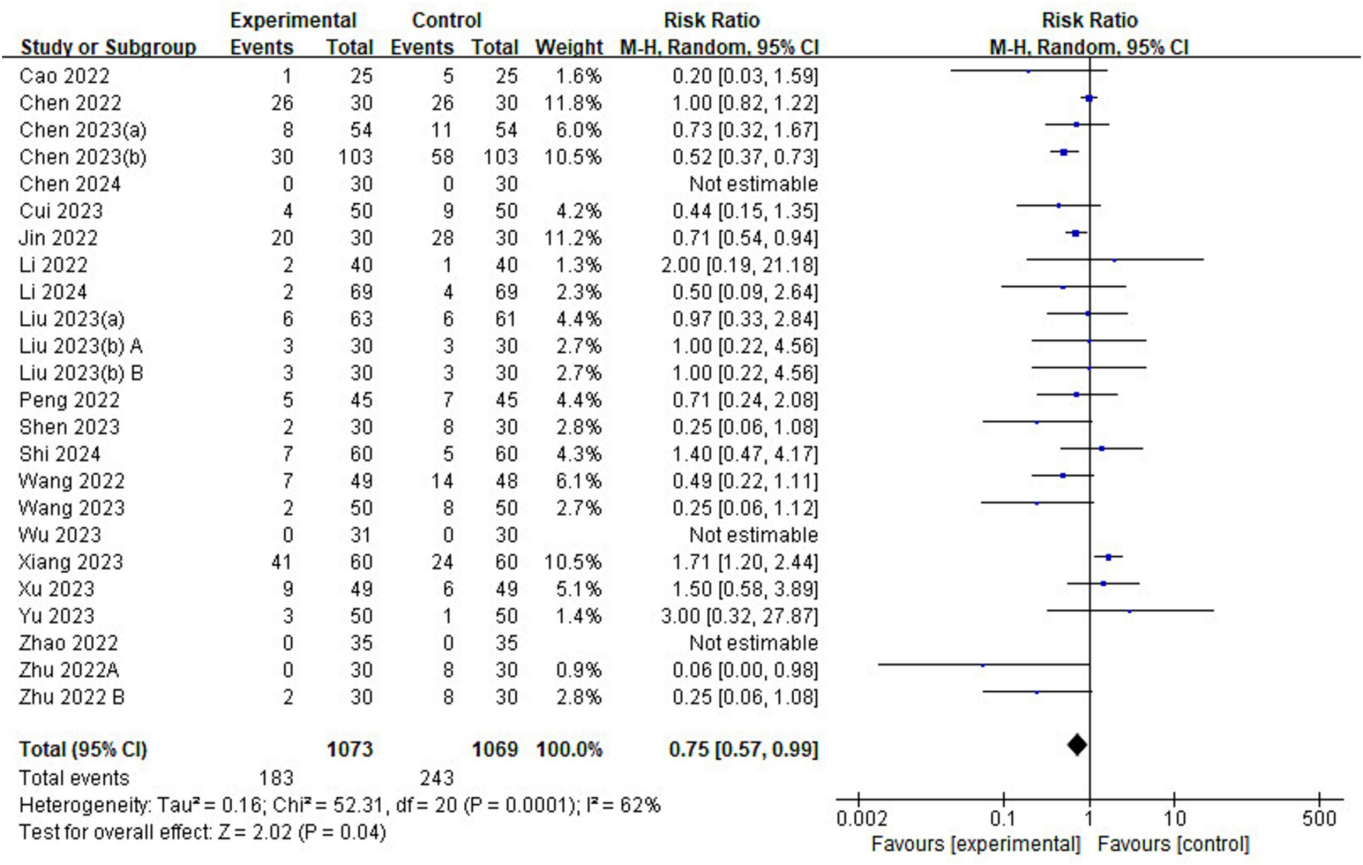
Forest plot comparing the risk of adverse events between esketamine and control groups. M-H, Mantel–Haenszel; CI, confidence interval.

Of the 23 included studies, and 22 reported details on the incidence of adverse events (Figure 9). The risk of adverse events was lower in the esketamine group than in the control group (RR = 0.75, 95% CI: [0.57–0.99], *p* = 0.04, *I*^2^ = 62%, 22 trials, 2,142 participants), suggesting that esketamine has some advantages in reducing the incidence of postoperative adverse events in children.

### 3.5 Publication bias and sensitivity analysis

**Figure 10:**
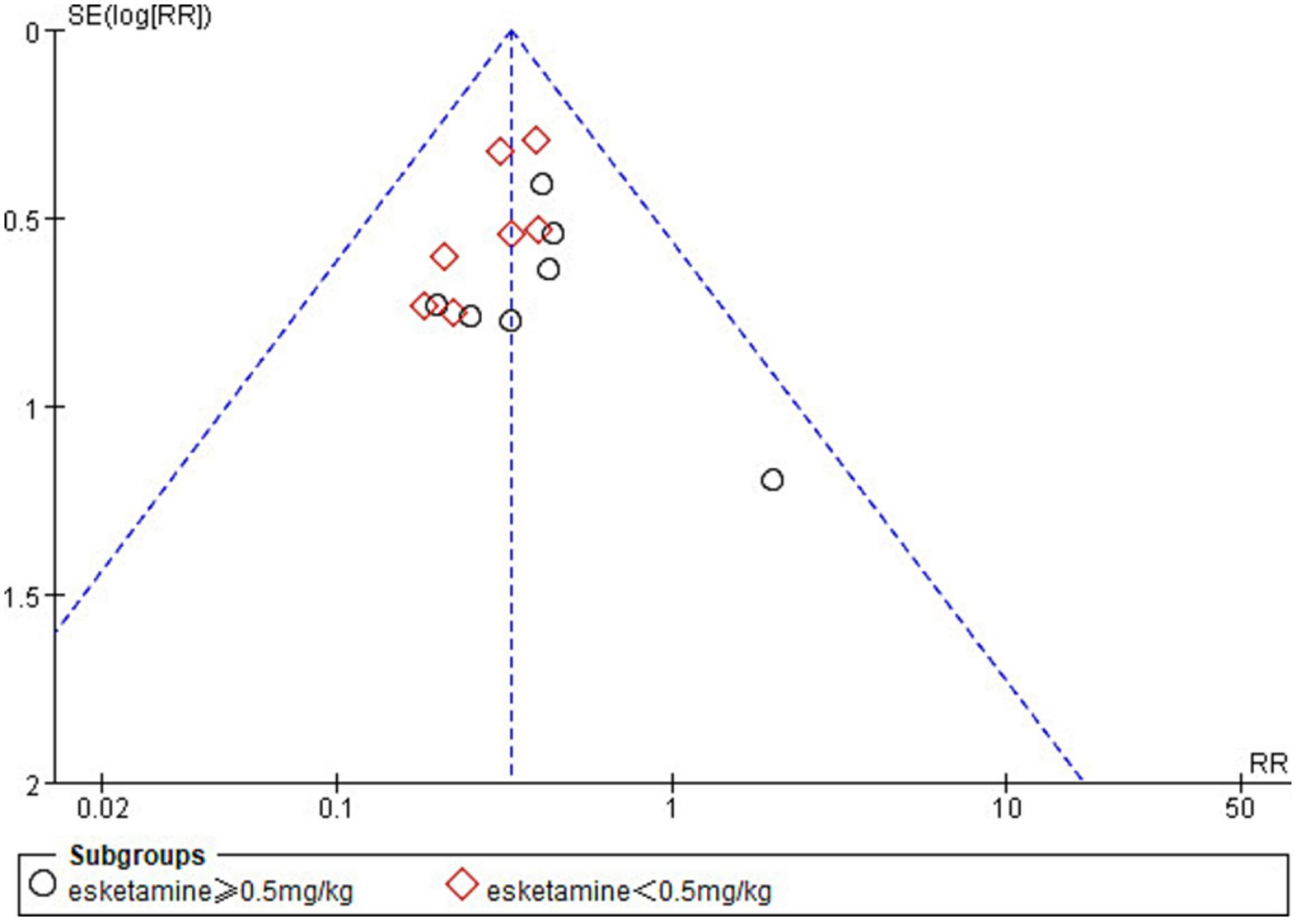
Funnel plot of the incidence of emergence delirium between esketamine and control groups.

**Figure 11:**
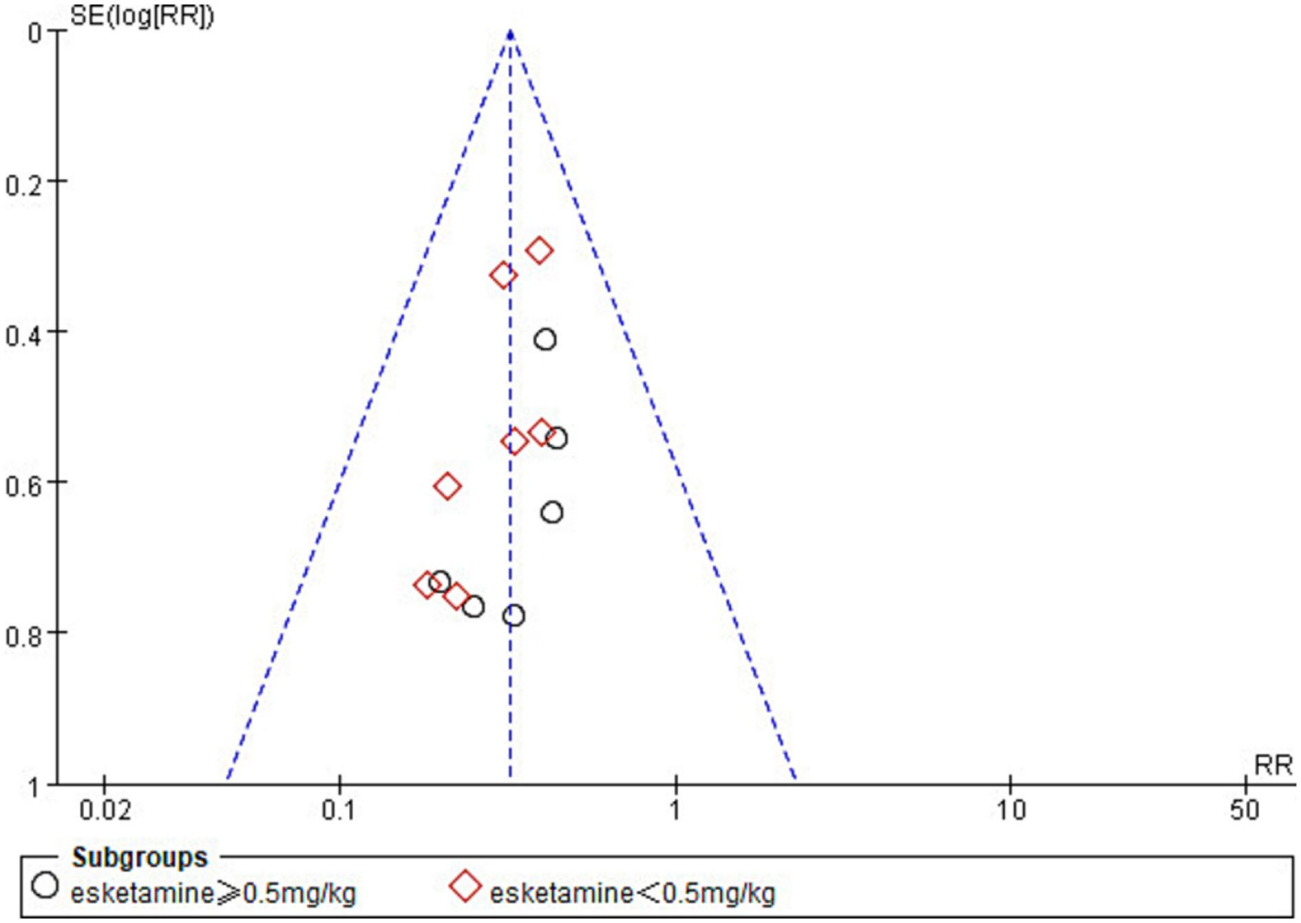
Funnel plot comparing the incidence of emergence delirium between esketamine and control groups. The funnel plot removes one study with high bias.

In the analysis of the incidence of ED, one study (42) was found to be at high risk of publication bias by plotting a funnel plot (Figure 10). After excluding the study with high risk of publication bias, the results showed that the funnel plot was symmetrical and suggested that publication bias was small (Figure 11).

After excluding each study, the effect of esketamine on the incidence of ED remained significant (RR = 0.32–0.34, 95% CI: [0.24–0.46]). The results showed studies with a high risk of bias did not unduly affect the pooled results (Table 4). This further strengthened the reliability of our conclusion that perioperative use of esketamine could reduce the incidence of ED in children undergoing tonsillectomy and adenoidectomy.

**Table 4:** Sensitivity analysis.

Excluded study ID	Sample size	P for *Q* test	*I*^2^ (%)	Effect mode	RR (95% CI)	P for *Z* test
None	1,113	0.93	0	FE	0.33 [0.25, 0.44]	<0.00001
Chen 2023(a)	1,005	0.93	0	FE	0.33 [0.25, 0.44]	<0.00001
Chen 2024	1,053	0.94	0	FE	0.33 [0.25, 0.43]	<0.00001
Jin 2022	1,053	0.94	0	FE	0.33 [0.25, 0.43]	<0.00001
Jin 2024	1,033	0.95	0	FE	0.34 [0.26, 0.45]	<0.00001
Li 2022	1,033	0.96	0	FE	0.34 [0.26, 0.45]	<0.00001
Liu 2023(b)	993	0.93	0	FE	0.34 [0.25, 0.45]	<0.00001
Shen 2023	1,053	0.93	0	FE	0.33 [0.25, 0.44]	<0.00001
Shi 2024	993	0.94	0	FE	0.32 [0.24, 0.43]	<0.00001
Wang 2022	1,016	0.91	0	FE	0.34 [0.26, 0.45]	<0.00001
Wu 2023	1,052	0.99	0	FE	0.32 [0.25, 0.43]	<0.00001
Xu 2023	1,015	0.95	0	FE	0.32 [0.24, 0.44]	<0.00001
Yu 2023	1,013	0.94	0	FE	0.34 [0.26, 0.44]	<0.00001
Zhao 2022	1,043	0.94	0	FE	0.34 [0.25, 0.46]	<0.00001

CI, confidence interval; RR, risk ratio; FE, fixed effect.

## 4 Discussion

Emergence delirium (ED) is an early complication of general anesthesia in pediatrics, presenting with perceptual deficits and psychomotor agitation (2). ED may cause postoperative complications, prolong hospital stay, and increase medical costs. The mechanism of ED in children remains unclear, and the risk factors of ED may include preschool age, ophthalmological and otorhinolaryngological procedures, inhalational anesthetics with low blood gas partition coefficients, prolonged duration of surgery, preoperative anxiety, and postoperative pain (43). The incidence of ED was significantly higher in pediatrics undergoing otorhinolaryngology procedures than in other pediatrics (44). The effect of different perioperative anesthetic drug use on emergence delirium also varies. However, perioperative use of ketamine may reduce the risk of ED (45, 46). Esketamine is the dextro isomer of ketamine and its pharmacological characteristics are similar to those of ketamine. However, compared to ketamine, esketamine has a stronger receptor affinity, stronger analgesic effect, faster metabolism, and fewer and milder adverse effects (11). Therefore, esketamine is widely used for the induction and maintenance of general anesthesia, ambulatory surgery, pediatric surgery, and postoperative analgesia.

In this systematic review and meta-analysis, we identified 23 randomized controlled trials that examined the effect of perioperative esketamine use on the incidence of delirium, delirium scores, pain scores, time to extubation, length of stay in the PACU, and incidence of adverse events in children undergoing tonsillectomy and adenoidectomy. Our results suggested that perioperative use of esketamine could reduce the risk of ED after general anesthesia when compared with a blank control or saline group, opioids, or other anesthetic drugs. Meanwhile, esketamine administrations before anesthesia, during anesthesia, or at the end of surgery could significantly reduce the incidence of ED. In addition, perioperative use of both anesthetic and subanaesthetic doses of esketamine significantly reduced the incidence of ED. Our study also found that perioperative use of esketamine reduced delirium and pain scores, shortened the length of stay in PACU, and reduced the risk of adverse events, but had little effect on the time to extubation.

ED is a common postoperative adverse event and prevention of ED is necessary (47). Our findings support the notion that perioperative use of esketamine could prevent ED. The ability of esketamine to reduce the risk of pediatric ED may be related to its unique pharmacological properties. Esketamine produces anesthesia and analgesia mainly through the inhibition of NMDA receptors and also produces analgesia through the inhibition of opioid receptors via G-protein coupling (48). While, one of the included studies had different results. The study of Wu et al. (42) reported there was no difference in the incidence of ED between the esketamine group and control group, which may be due to the administration of remimazolam in the the control group. Remazolam is a new ultra-short-acting benzodiazepine that can relieve preoperative anxiety. In addition, Yang et al. (49) found that administration of remimazolam at the end of the surgery could reduce the incidence of ED in children following tonsillectomy and adenoidectomy under sevoflurane anesthesia.

Three of the included studies were esketamine versus other anesthetic drugs (20, 41, 42). The control groups in these three studies were administrated remimazolam, midazolam, and dexmedetomidine, respectively. Although we found that esketamine reduced the risk of ED compared with midazolam and dexmedetomidine, there was only one study with a small sample size to support this result, respectively. Further multi-center, large sample-size clinical trials are needed to determine the effect of esketamine on ED compared to other anesthetics. Compared with the blank control or saline group, esketamine significantly reduced the incidence of ED. This suggests that adding esketamine to the standard anesthetic regimen may help prevent ED. However, Chen et al.’s findings were contrary, showing that a single dose of near-anesthetic for anesthesia induction may increase the risk of ED in preschool children after surgery (50). This may be due to a higher proportion of children in the esketamine group who were treated with sevofluorine for maintenance of anesthesia. In order to determine the effect of perioperative esketamine use on ED, more standardized anesthetic regimens are necessary in the future. In summary, based on our findings, it can be inferred that perioperative esketamine use could prevent ED, but further evidence from higher-quality studies is needed.

Pain is an important risk factor for ED in children. Esketamine could provide effective analgesia, reduce postoperative pain scores, decrease opioid consumption, and improve the quality of perioperative recovery (51, 52). This meta-analysis showed that esketamine can reduce postoperative pain levels, which is consistent with the findings of Qian et al. (53). Five of the included studies showed that perioperative use of esketamine reduced postoperative pain in children undergoing tonsillectomy and adenoidectomy compared with perioperative use of opioids and one of the included studies showed that esketamine was comparable to opioids for analgesia (28). These results suggest that esketamine is effective in improving postoperative pain in children undergoing tonsillectomy and adenoidectomy. Heavy opioid use can cause pain hypersensitivity, which is a state of hypersensitivity to painful stimuli (54). However, esketamine could relieve opioid-induced pain hypersensitivity and enhance opioid analgesia (55).

It is important to note that doses of perioperative esketamine use vary (from 0.15 mg/kg to 1 mg/kg). Meanwhile, times of esketamine administration are also different (including before anesthesia, during anesthesia, and at the end of surgery). Although this meta-analysis showed that different times of administration of esketamine and different doses of esketamine both could reduce the incidence of ED, we are unable to simultaneously determine an optimal timing and dosage of administration to prevent ED. The pharmacological effect of esketamine at different doses and times may be different in extent and duration, which may have different effects on results. The difference in the dose and time of esketamine administration between studies may also introduce confounding factors and bias the results.

This systematic review and meta-analysis still has several potential limitations. Firstly, most of the studies included were small studies with a sample size of each group less than 100, which may lead to small effect study bias (56). Second, ED is delirium that occurs in the operating room or PACU after anesthesia has ended. However, the time point of assessing ED was inconsistent among the included studies which may lead to inconsistent measurement results. Third, the usage and dosage of esketamine varied among the included studies, and we are unable to provide valuable recommendations for the use of esketamine in the perioperative period. Fourth, The measurement tools were different in included studies. As a result, there will be some differences in the results. Therefore, further more standardized perioperative esketamine protocols and unified assessment tools for ED should be developed for prevention of ED in children undergoing tonsillectomy and adenoidectomy. Fifth, there are data source limitations in this study, and all included studies were conducted in China. This may affect the external validity of the results due to differences in medical practice, patient characteristics, anesthesia management practices, and so on in different countries. More international studies are needed in the future, including groups of children in different regions, to validate our findings and improve the broad applicability of the results.

## 5 Conclusion

In conclusion, this systematic review and meta-analysis suggests that the perioperative use of esketamine could significantly reduce the incidence of ED in children undergoing tonsillectomy and adenoidectomy. In addition, perioperative administration of esketamine reduces the risk of postoperative adverse events. However, the optimal dose and timing of esketamine administration for preventing ED remains to be explored.

## Data Availability

The original contributions presented in the study are included in the article/Supplementary material, further inquiries can be directed to the corresponding author.

## Supplementary material

SUPPLEMENTARY FIGURE 1 Forest plot comparing the risk of emergence delirium between esketamine and different control groups. M-H, Mantel-Haenszel; CI, confidence interval.

SUPPLEMENTARY FIGURE 2 Forest plot comparing the risk of emergence delirium between different doses of esketamine and control groups. M-H, Mantel-Haenszel; CI, confidence interval.

SUPPLEMENTARY FIGURE 3 Forest plot comparing the risk of emergence delirium between esketamine administrated at different time and control groups. M-H, Mantel-Haenszel; CI, confidence interval.
