# Supplementary figures and images for: Effect of perioperative esketamine use on emergency delirium in children undergoing tonsillectomy and adenoidectomy: a systematic review and meta-analysis of randomized controlled trials
Source: Front Med (Lausanne). 2025 Jan 29;12:1505408. doi: 10.3389/fmed.2025.1505408 (PMC11814166; doi:10.3389/fmed.2025.1505408)

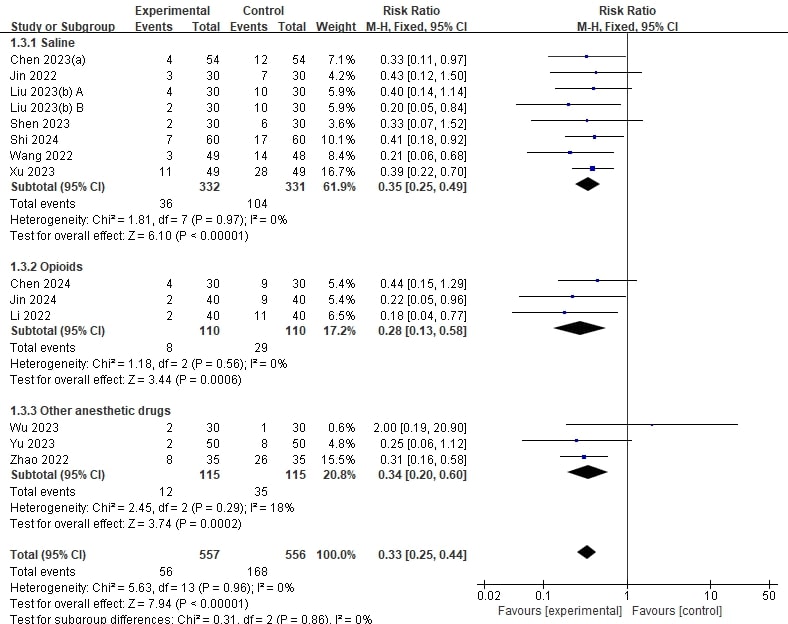

Supplement: SUPPLEMENTARY FIGURE 1 — Forest plot comparing the risk of emergence delirium between esketamine and different control groups. M-H, Mantel-Haenszel; CI, confidence interval. [file Image_1.JPEG]

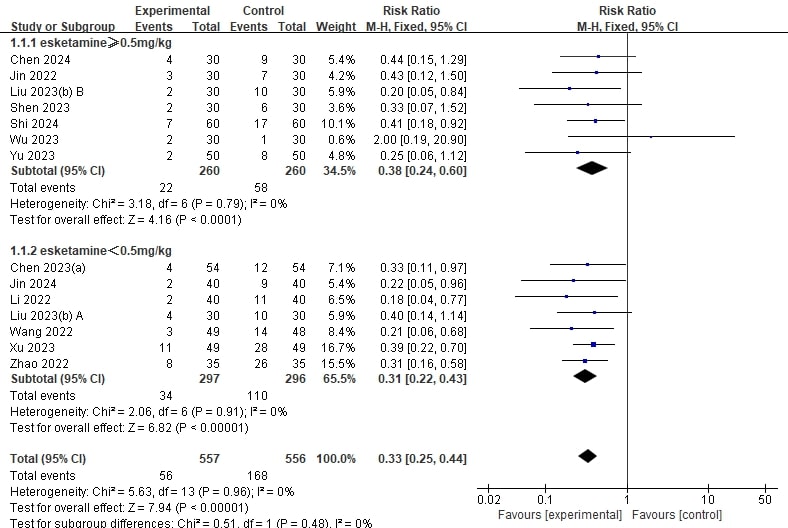

Supplement: SUPPLEMENTARY FIGURE 2 — Forest plot comparing the risk of emergence delirium between different doses of esketamine and control groups. M-H, Mantel-Haenszel; CI, confidence interval. [file Image_2.JPEG]

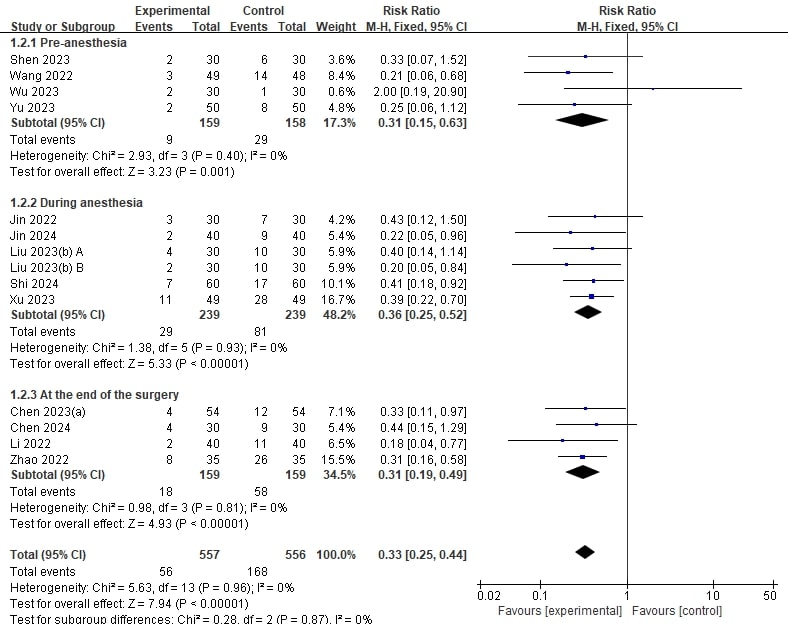

Supplement: SUPPLEMENTARY FIGURE 3 — Forest plot comparing the risk of emergence delirium between esketamine administrated at different time and control groups. M-H, Mantel-Haenszel; CI, confidence interval. [file Image_3.JPEG]
