# Supplementary material for: Effect of perioperative esketamine use on emergency delirium in children undergoing tonsillectomy and adenoidectomy: a systematic review and meta-analysis of randomized controlled trials
Source: Front Med (Lausanne). 2025 Jan 29;12:1505408. doi: 10.3389/fmed.2025.1505408 (PMC11814166; doi:10.3389/fmed.2025.1505408)
Supplement: Supplementary file 4 [file Table_1.DOCX]

**2024.9.1**

Embase3+Cochrane15+Web of science2+ Medline2+pubmed17+sinomed 13+Wangfang21 + CNKI 19+VIP16=108

**Embase**

| #5 | 3 | #1 AND #2AND #3AND #4 |
| --- | --- | --- |
| #4 | 2160382 | **'pediatrics':ti,ab,kw OR 'children':ti,ab,kw OR 'child':ti,ab,kw** |
| #3 | 15038 | tonsillectomy:ti,ab,kw OR adenoidectomy:ti,ab,kw |
| #2 | 2487 | **'am 101':ti,ab,kw OR 'am101':ti,ab,kw OR 'cle 100':ti,ab,kw OR 'cle100':ti,ab,kw OR 'cz 06':ti,ab,kw OR 'cz06':ti,ab,kw OR 'esgamda':ti,ab,kw OR 'eskelan':ti,ab,kw OR 'esketamin':ti,ab,kw OR 'esketamine hydrochloride':ti,ab,kw OR 'esketiv':ti,ab,kw OR 'falkieri':ti,ab,kw OR 'jnj 54135419':ti,ab,kw OR 'jnj 5419':ti,ab,kw OR 'jnj54135419':ti,ab,kw OR 'jnj5419':ti,ab,kw OR 'ketanest s':ti,ab,kw OR 'keyzilen':ti,ab,kw OR 'pg 061':ti,ab,kw OR 'pg061':ti,ab,kw OR 's ketamin':ti,ab,kw OR 's ketamine':ti,ab,kw OR 's-ketamin':ti,ab,kw OR 'sinmelan':ti,ab,kw OR 'spravato':ti,ab,kw OR 'vesierra':ti,ab,kw OR 'esketamine':ti,ab,kw** |
| #1 | 1498 | 'agitated emergence':ti,ab,kw OR 'emergence delirium':ti,ab,kw OR 'emergence excitement':ti,ab,kw OR 'post-anaesthetic agitation':ti,ab,kw OR 'post-anaesthetic delirium':ti,ab,kw OR 'post-anaesthetic excitement':ti,ab,kw OR 'post-anesthetic agitation':ti,ab,kw OR 'post-anesthetic delirium':ti,ab,kw OR 'post-anesthetic excitement':ti,ab,kw OR 'postanaesthetic agitation':ti,ab,kw OR 'postanaesthetic delirium':ti,ab,kw OR 'postanaesthetic excitement':ti,ab,kw OR 'postanesthetic agitation':ti,ab,kw OR 'postanesthetic delirium':ti,ab,kw OR 'postanesthetic excitement':ti,ab,kw OR 'emergence agitation':ti,ab,kw |

**Cochrane 15**

Esketamine or S-Ketamine or L-Ketamine or Kataved or Spravato in Title Abstract Keyword AND Agitated Emergence or Delirium, Post-Operative or Agitation, Emergence or Postanesthetic Excitement or Emergence Agitation or Emergence, Agitated or Agitations, Emergence or Emergence Excitement or Excitement, Emergence or Delirium, Postoperative or Postoperative Delirium or Post-Operative Delirium or Excitement, Postanesthetic or Post Operative Delirium or Anesthesia Emergence Delirium or Delirium, Emergence or Delirium, Anesthesia Emergence or Emergence Delirium, Anesthesia in Title Abstract Keyword AND Children or Pediatrics in Title Abstract Keyword AND tonsillectomy or adenoidectomy in Title Abstract Keyword - (Word variations have been searched)

**Web of science**

| #5 | 2 | #1 AND #2AND #3AND #4 |
| --- | --- | --- |
| #4 | 1763419 | **(TS=(Pediatrics)) OR TS=(Children)** |
| #3 | 9750 | (((TS=(Tonsillectomy)) OR TS=(Tonsillectomies)) OR TS=(Adenoidectomy)) OR TS=(Adenoidectomies) |
| #2 | 2010 | **((((TS=(Esketamine)) OR TS=(L-Ketamine)) OR TS=(S-Ketamine)) OR TS=(Spravato)) OR TS=((S)-2-(o-chlorophenyl)-2-(methylamino)cyclohexanone)** |
| #1 | 7751 | **(((((((((TS=(Emergence delirium )) OR TS=(Agitated Emergence))) OR TS=(Emergence Agitation)) OR TS=(Emergence Excitement)) OR TS=(Postanesthetic Excitement)) OR TS=(Anesthesia Emergence Delirium)) OR TS=(Postoperative Delirium)) OR TS=(Post-Operative Delirium)) OR TS=(Post Operative Delirium)** |

**Medline**

| #5 | 2 | #1 AND #2AND #3AND #4 |
| --- | --- | --- |
| #4 | 2770877 | **(TS=(Pediatrics)) OR TS=(Children)** |
| #3 | 15750 | (((TS=(Tonsillectomy)) OR TS=(Tonsillectomies)) OR TS=(Adenoidectomy)) OR TS=(Adenoidectomies) |
| #2 | 1783 | **((((TS=(Esketamine)) OR TS=(L-Ketamine)) OR TS=(S-Ketamine)) OR TS=(Spravato)) OR TS=((S)-2-(o-chlorophenyl)-2-(methylamino)cyclohexanone)** |
| #1 | 6808 | **(((((((((TS=(Emergence delirium )) OR TS=(Agitated Emergence))) OR TS=(Emergence Agitation)) OR TS=(Emergence Excitement)) OR TS=(Postanesthetic Excitement)) OR TS=(Anesthesia Emergence Delirium)) OR TS=(Postoperative Delirium)) OR TS=(Post-Operative Delirium)) OR TS=(Post Operative Delirium)** |

**Pubmed**

| #5 | 17 | #1 AND #2AND #3AND #4   Search: #1 AND #2AND #3AND #4  ("emergence delirium"[MeSH Terms] OR ("emergence delirium"[MeSH Terms] OR ("emergence"[All Fields] AND "delirium"[All Fields]) OR "emergence delirium"[All Fields] OR ("agitated"[All Fields] AND "emergence"[All Fields]) OR "agitated emergence"[All Fields]) OR ("emergence delirium"[MeSH Terms] OR ("emergence"[All Fields] AND "delirium"[All Fields]) OR "emergence delirium"[All Fields] OR ("emergence"[All Fields] AND "agitation"[All Fields]) OR "emergence agitation"[All Fields]) OR ("emergence delirium"[MeSH Terms] OR ("emergence"[All Fields] AND "delirium"[All Fields]) OR "emergence delirium"[All Fields] OR ("emergence"[All Fields] AND "excitement"[All Fields]) OR "emergence excitement"[All Fields]) OR ("emergence delirium"[MeSH Terms] OR ("emergence"[All Fields] AND "delirium"[All Fields]) OR "emergence delirium"[All Fields] OR ("postanesthetic"[All Fields] AND "excitement"[All Fields]) OR "postanesthetic excitement"[All Fields]) OR ("emergence delirium"[MeSH Terms] OR ("emergence"[All Fields] AND "delirium"[All Fields]) OR "emergence delirium"[All Fields] OR ("anesthesia"[All Fields] AND "emergence"[All Fields] AND "delirium"[All Fields]) OR "anesthesia emergence delirium"[All Fields]) OR ("emergence delirium"[MeSH Terms] OR ("emergence"[All Fields] AND "delirium"[All Fields]) OR "emergence delirium"[All Fields] OR ("postoperative"[All Fields] AND "delirium"[All Fields]) OR "postoperative delirium"[All Fields]) OR ("emergence delirium"[MeSH Terms] OR ("emergence"[All Fields] AND "delirium"[All Fields]) OR "emergence delirium"[All Fields] OR ("post"[All Fields] AND "operative"[All Fields] AND "delirium"[All Fields]) OR "post operative delirium"[All Fields]) OR ("emergence delirium"[MeSH Terms] OR ("emergence"[All Fields] AND "delirium"[All Fields]) OR "emergence delirium"[All Fields] OR ("post"[All Fields] AND "operative"[All Fields] AND "delirium"[All Fields]) OR "post operative delirium"[All Fields])) AND ("esketamine"[Supplementary Concept] OR "esketamine"[All Fields] OR "l ketamine"[All Fields] OR ("esketamine"[Supplementary Concept] OR "esketamine"[All Fields] OR "ketamine"[All Fields] OR "ketamine"[MeSH Terms] OR "ketamin"[All Fields] OR "ketamine s"[All Fields] OR "ketamines"[All Fields]) OR ("esketamine"[Supplementary Concept] OR "esketamine"[All Fields] OR "s ketamine"[All Fields]) OR ((((("S"[All Fields] AND "2"[All Fields]) AND "o-chlorophenyl"[All Fields]) AND "2"[All Fields]) AND "methylamino"[All Fields]) AND ("cyclohexanone"[Supplementary Concept] OR "cyclohexanone"[All Fields] OR "cyclohexanones"[MeSH Terms] OR "cyclohexanones"[All Fields])) OR ("esketamine"[Supplementary Concept] OR "esketamine"[All Fields]) OR ("esketamine"[Supplementary Concept] OR "esketamine"[All Fields] OR "esketamine"[All Fields] OR "spravato"[All Fields])) AND ("pediatrics"[MeSH Terms] OR "child"[MeSH Terms] OR ("child"[MeSH Terms] OR "child"[All Fields] OR "children"[All Fields] OR "child s"[All Fields] OR "children s"[All Fields] OR "childrens"[All Fields] OR "childs"[All Fields])) AND ("tonsillectomy"[MeSH Terms] OR ("tonsillectomy"[MeSH Terms] OR "tonsillectomy"[All Fields] OR "tonsillectomies"[All Fields]) OR "adenoidectomy"[MeSH Terms] OR ("adenoidectomy"[MeSH Terms] OR "adenoidectomy"[All Fields] OR "adenoidectomies"[All Fields])) |
| --- | --- | --- |
| #4 | 15770 | #4"tonsillectomy"[MeSH Terms] OR "tonsillectomy"[MeSH Terms] OR "tonsillectomy"[All Fields] OR "tonsillectomies"[All Fields] OR "adenoidectomy"[MeSH Terms] OR "adenoidectomy"[MeSH Terms] OR "adenoidectomy"[All Fields] OR "adenoidectomies"[All Fields] |
| #3 | [3293700](https://pubmed.ncbi.nlm.nih.gov/?term=((pediatrics[MeSH+Terms])+OR+(child[MeSH+Terms]))+OR+(children)&sort=) | #3"pediatrics"[MeSH Terms] OR "child"[MeSH Terms] OR "child"[MeSH Terms] OR "child"[All Fields] OR "children"[All Fields] OR "child s"[All Fields] OR "children s"[All Fields] OR "childrens"[All Fields] OR "childs"[All Fields] |
| #2 | 26002 | #2"esketamine"[Supplementary Concept] OR "esketamine"[All Fields] OR "l ketamine"[All Fields] OR ("esketamine"[Supplementary Concept] OR "esketamine"[All Fields] OR "ketamine"[All Fields] OR "ketamine"[MeSH Terms] OR "ketamin"[All Fields] OR "ketamine s"[All Fields] OR "ketamines"[All Fields]) OR ("esketamine"[Supplementary Concept] OR "esketamine"[All Fields] OR "s ketamine"[All Fields]) OR ((((("S"[All Fields] AND "2"[All Fields]) AND "o-chlorophenyl"[All Fields]) AND "2"[All Fields]) AND "methylamino"[All Fields]) AND ("cyclohexanone"[Supplementary Concept] OR "cyclohexanone"[All Fields] OR "cyclohexanones"[MeSH Terms] OR "cyclohexanones"[All Fields])) OR ("esketamine"[Supplementary Concept] OR "esketamine"[All Fields]) OR ("esketamine"[Supplementary Concept] OR "esketamine"[All Fields] OR "esketamine"[All Fields] OR "spravato"[All Fields]) |
| #1 | 6834 | #1"emergence delirium"[MeSH Terms] OR ("emergence delirium"[MeSH Terms] OR ("emergence"[All Fields] AND "delirium"[All Fields]) OR "emergence delirium"[All Fields] OR ("agitated"[All Fields] AND "emergence"[All Fields]) OR "agitated emergence"[All Fields]) OR ("emergence delirium"[MeSH Terms] OR ("emergence"[All Fields] AND "delirium"[All Fields]) OR "emergence delirium"[All Fields] OR ("emergence"[All Fields] AND "agitation"[All Fields]) OR "emergence agitation"[All Fields]) OR ("emergence delirium"[MeSH Terms] OR ("emergence"[All Fields] AND "delirium"[All Fields]) OR "emergence delirium"[All Fields] OR ("emergence"[All Fields] AND "excitement"[All Fields]) OR "emergence excitement"[All Fields]) OR ("emergence delirium"[MeSH Terms] OR ("emergence"[All Fields] AND "delirium"[All Fields]) OR "emergence delirium"[All Fields] OR ("postanesthetic"[All Fields] AND "excitement"[All Fields]) OR "postanesthetic excitement"[All Fields]) OR ("emergence delirium"[MeSH Terms] OR ("emergence"[All Fields] AND "delirium"[All Fields]) OR "emergence delirium"[All Fields] OR ("anesthesia"[All Fields] AND "emergence"[All Fields] AND "delirium"[All Fields]) OR "anesthesia emergence delirium"[All Fields]) OR ("emergence delirium"[MeSH Terms] OR ("emergence"[All Fields] AND "delirium"[All Fields]) OR "emergence delirium"[All Fields] OR ("postoperative"[All Fields] AND "delirium"[All Fields]) OR "postoperative delirium"[All Fields]) OR ("emergence delirium"[MeSH Terms] OR ("emergence"[All Fields] AND "delirium"[All Fields]) OR "emergence delirium"[All Fields] OR ("post"[All Fields] AND "operative"[All Fields] AND "delirium"[All Fields]) OR "post operative delirium"[All Fields]) OR ("emergence delirium"[MeSH Terms] OR ("emergence"[All Fields] AND "delirium"[All Fields]) OR "emergence delirium"[All Fields] OR ("post"[All Fields] AND "operative"[All Fields] AND "delirium"[All Fields]) OR "post operative delirium"[All Fields]) |

**Sinomed 13**

("苏醒谵妄"[全部字段] OR "Emergence Delirium"[全部字段] OR "苏醒兴奋"[全部字段] OR "麻醉后兴奋"[全部字段] OR "麻醉苏醒谵妄"[全部字段] OR "苏醒期躁动"[全部字段] OR "苏醒谵妄"[主题词]) AND "艾司氯胺酮"[全部字段] AND (("扁桃体切除术"[全部字段] OR "Tonsillectomy"[全部字段] OR "扁桃体切除术"[主题词]) OR ("腺样体切除术"[全部字段] OR "Adenoidectomy"[全部字段] OR "腺样体切除术"[主题词])) AND ("儿童"[全部字段] OR "Child"[全部字段] OR "儿童"[主题词])

**VIP** **16**

U=（苏醒谵妄 + 麻醉苏醒谵妄 + 苏醒兴奋 + 麻醉后兴奋 + 苏醒期躁动 + 术后躁动) AND U=(艾司氯胺酮) AND U=(扁桃体切除术 + 腺样体切除术) AND U=(小儿 + 儿童)

**Wangfang 21**

主题:(苏醒谵妄 or 麻醉苏醒谵妄 or 苏醒兴奋 or 麻醉后兴奋 or 苏醒期躁动 or 术后躁动 ) and 主题:(艾司氯胺酮) and 主题:(扁桃体切除术 or 腺样体切除术) and 主题:(儿童 or 小儿 or 患儿)

**CNKI 19**

FT % '苏醒谵妄' + '麻醉苏醒谵妄' + '苏醒兴奋' + '麻醉后兴奋' + '苏醒期躁动' AND TKA % '艾司氯胺酮' AND TKA % '扁桃体切除术' + '腺样体切除术' AND TKA % '小儿' + '儿童' + '患儿'
